# The diagnostic value of the neurological examination in coma of unknown etiology

**DOI:** 10.1007/s00415-021-10527-4

**Published:** 2021-04-01

**Authors:** Wolf U. Schmidt, M. Lutz, C. J. Ploner, M. Braun

**Affiliations:** 1 Department of Neurology, Charité Universitätsmedizin Berlin, Corporate Member of Freie Universität Berlin, Humboldt-Universität zu Berlin, Berlin Institute of Health, Campus Virchow-Klinikum, Augustenburger Platz 1, 13353 Berlin, Germany; 2 Center for Stroke Research, Charité Universitätsmedizin Berlin, Corporate Member of Freie Universität Berlin, Humboldt-Universität zu Berlin, Berlin Institute of Health, Charitéplatz 1, Berlin, 10117 Germany

**Keywords:** Coma, Neurologic examination, Reproducibility of results, Neurological emergencies, Critical pathways

## Abstract

**Background:**

Identifying the cause of non-traumatic coma in the emergency department is challenging. The clinical neurological examination is the most readily available tool to detect focal neurological deficits as indicators for cerebral causes of coma. Previously proposed clinical pathways have granted the interpretation of clinical findings a pivotal role in the diagnostic work-up. We aimed to identify the actual diagnostic reliability of the neurological examination with regard to identifying acute brain damage.

**Methods:**

Eight hundred and fifty-three patients with coma of unknown etiology (CUE) were examined neurologically in the emergency department following a predefined routine. Coma-explaining pathologies were identified retrospectively and grouped into primary brain pathology with proof of acute brain damage and other causes without proof of acute structural pathology. Sensitivity, specificity and percentage of correct predictions of different examination protocols were calculated using contingency tables and binary logistic regression models.

**Results:**

The full neurological examination was 74% sensitive and 60% specific to detect acute structural brain damage underlying CUE. Sensitivity and specificity were higher in non-sedated patients (87/61%) compared to sedated patients (64%/59%). A shortened four-item examination protocol focusing on pupils, gaze and pyramidal tract signs was only slightly less sensitive (67%) and more specific (65%).

**Conclusions:**

Due to limited diagnostic reliability of the physical examination, the absence of focal neurological signs in acutely comatose patients should not defer from a complete work-up including brain imaging. In an emergency, a concise neurological examination should thus serve as one part of a multimodal diagnostic approach to CUE.

## Introduction

Coma of unknown etiology (CUE) is a challenging emergency. CUE defines an impairment of consciousness for reasons other than traumatic brain injury or cerebral hypoperfusion in cardiac arrest. Causes of CUE include neurological, neurosurgical and medical conditions many of which are time-sensitive and life-threatening emergencies [1, 2]. Early detection of acute structural brain damage is of the utmost importance as it may require referral for specialist treatment. Diagnostic tools available in the emergency department include history taking, clinical examination, imaging and laboratory methods. A neurological examination is often the first step in the diagnostic work-up of CUE patients and serves three purposes: First, the examiner must assess whether there is indeed an impairment of consciousness and if yes, what the degree of impairment is. Numerical scales for this purpose are the Glasgow Coma Scale (GCS) and the Full Outline of UnResponsiveness (FOUR) Score [3, 4]. Second, early neurological examination sets a baseline for assessing the patient’s functional status over time and thus critically influences diagnostics and treatment [5, 6]. Third, neurological examination is the most readily available diagnostic tool to identify the nature of the underlying disorder [6, 7]. Examination techniques have frequently been considered reliable tools to discriminate acute structural brain damage from other causes [5, 8, 9]. Accordingly, many guidelines for emergency management of CUE grant the interpretation of clinical findings a pivotal role in decision-making and determination of subsequent work-up [5, 10, 11]. However, despite the attributed importance, the predictive value of clinical findings in CUE patients has not been investigated so far. At least in awake patients, clinical neurological findings were shown to have limited sensitivity and specificity [12–15]. In addition, there is little doubt that clinical findings are associated with a significant interobserver variability [16–18].

The purpose of this study was to determine and quantify the predictive value of the neurological assessment with regard to detecting acute structural supra-tentorial or infra-tentorial brain damage as the cause of CUE in emergency patients. We aimed to identify those neurological examination findings that serve this purpose with high reliability. The significance of the clinical examination in the initial emergency management of CUE should be evaluated by empirical evidence.

## Methods

### Setting

Charité Campus Virchow-Klinikum is a tertiary care university hospital located in Berlin, Germany, caring for approximately 70,000 emergency patients per year.

### Patients/study size

We previously reported on *n* = 1027 prospectively recruited adult emergency patients presenting with CUE between May 2013 and January 2017 [2]. By the time of the first neurological assessment, 173 of them had spontaneously regained consciousness. The remaining 854 unconscious patients were examined neurologically immediately after the assessment of vital parameters and rapid ABC management.

Data were collected by neurologists in the emergency department who consulted the patients immediately, following a predefined management procedure [19]. Patients’ final diagnoses were unknown at the time of examination and added to their datasets after completion of diagnostic procedures.

### Examination protocol

**Fig. 1:**
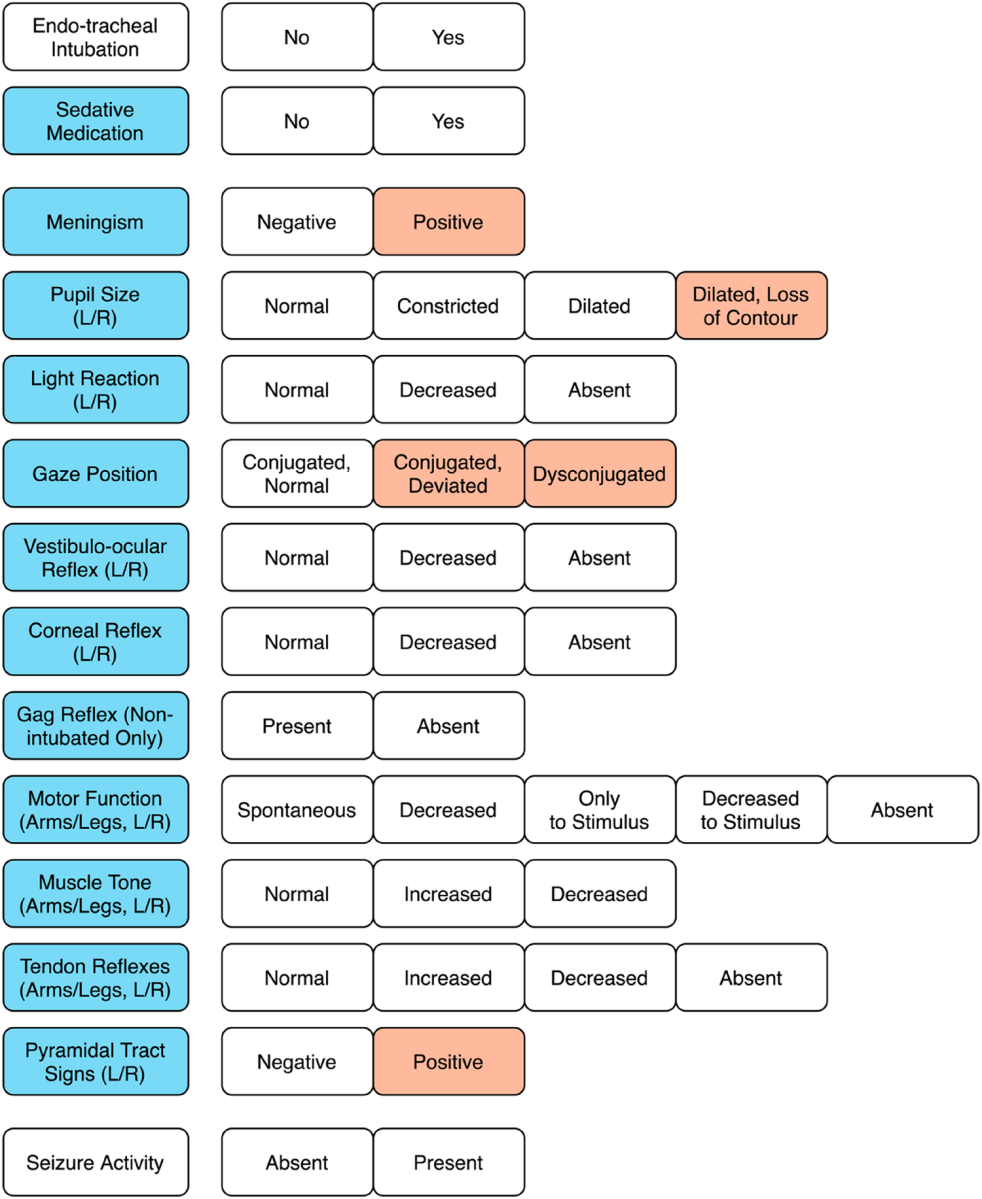
Examination protocol. First column: Items to be evaluated; subsequent columns: categorized results. Blue items were used to statistically evaluate the neurological assessment. An item was marked positive, i.e., pointing towards acute structural brain damage, whenever there were asymmetrical findings in a patient or when a finding was recorded regardless of asymmetry (red items)

Neurological examinations were performed according to a predefined protocol [19]. Results were documented electronically in the hospital’s patient information system. Neurologists determined the ability to communicate verbally or by eye, head or limb movements and quantified the level of consciousness by Glasgow Coma Scale (GCS) evaluation. We scored GCS values following a “rate-what-you-see”-principle (e.g., verbal score = 1 when intubated) to describe the level of consciousness in the real-life study cohort regardless of different etiologies. The examination protocol (Fig. 1, left) required examiners to assess the following items: intubation (yes/no), sedation (yes/no), meningism, pupil size, pupil reaction to light, position of gaze, vestibulo-ocular reflex, corneal reflex, gag reflex (in non-intubated patients only), movement of all limbs (incl. reaction to painful stimuli), muscle tone of all limbs, tendon reflexes of all limbs, pyramidal tract signs and the presence or absence of seizure activity. Clinical findings were documented as predefined categorized variables (Fig. 1, right) including optional customized additions.

### Emergency management/diagnoses

Emergency management of patients presenting with CUE followed standardized operating procedures [19]. After completion of diagnostics, in a retrospective analysis, each patient was given one main diagnosis explaining the disorder of consciousness as described earlier [2].

Summarized, two board-certified neurologists (WS, MB) reviewed all available clinical, laboratory, radiology and (if available) autopsy findings. Main diagnoses were from one of three categories: (I) primary brain pathology with proof of acute structural or inflammatory brain damage (e.g., bleeding, infarction, meningoencephalitis), (II) primary brain pathology without acute structural damage (e.g., epilepsy) or (III) acute systemic disorders affecting the brain secondarily (e.g., metabolic disorder, intoxication). Whenever acute structural brain damage was present and could explain the disorder of consciousness directly (by its location and/or size) or indirectly (e.g., by causing an acute symptomatic seizure), patients were given a main diagnosis from category I. This holds true even in the presence of coexisting further potential coma explaining pathologies from category III, e.g., in a patient with subdural hematoma and severe alcohol intoxication. If categorization of the underlying coma explaining pathology was divergent between the two investigators, a detailed re-evaluation and discussion of patient data were performed until consensus was reached. Table 1 shows the distribution of all final diagnoses in the study cohort.

**Table 1:** Distribution of final diagnoses in the study cohort

	Diagnosis	Patients
I	Intracranial hemorrhage	190
	Infarction	95
	Inflammation	22
	Tumor	17
	Other primary CNS damage	6
II	Epilepsy	188
	Neuro-degenerative disease	4
	Psychiatric disease	21
	Cardiac/pulmonary	53
III	Metabolic/homeostatic	50
	Septic encephalopathy	25
	Intoxication	164
	Other secondary CNS affection	2
	Surgical emergency	6
	Unspecified secondary CNS Affection	10

The predictive value of the neurological examination was defined as its ability to predict acute structural brain damage (category I, above the line) from all other pathologies causing disorders of consciousness (categories II and III, below the line)

### Data preparation

Digitalized clinical findings, diagnoses and adjacent variables were extracted from the patients’ files and anonymized. For one out of 854 patients, no examination was documented. This dataset was thus excluded from analysis. All asymmetrical findings (i.e., findings that were variable between left and right sides on direct comparison including dysconjugated and/or deviated position of gaze) were considered suggestive of primary structural brain pathology (“red flag”). Exceptions from this rule were extreme bilateral dilatation of pupils with loss of contour which was interpreted as a sign of elevated intracranial pressure until proven otherwise and positive pyramidal tract signs being flagged as suspicious when present unilaterally *or* bilaterally. Pathological flexion or extension responses were always flagged as indicators of CNS (namely midbrain or brainstem) pathology. Meningism (neck rigidity, Kernig’s or Brudzinski’s Sign, was considered suspicious when positive. Gag reflex in non-intubated patients was considered suspicious when absent regardless of lateralization because we did not expect a reliable bilateral assessment in the emergency. The presence of seizure activity in CUE was considered suspicious for acute structural brain pathology when it appeared in conjunction with other neurological deficits present before or after the seizure. Seizure activity alone served as an indicator for primary brain pathology without acute structural damage such as epilepsy (other than acute symptomatic seizures) [2].

### Data analysis

We retrospectively calculated the neurological examination’s sensitivity and specificity to detect acute structural brain pathology causing CUE. We applied various filters to the data and performed different calculations for (A) the whole examination including all items (and thus all possible “red flags”), (B) single items of the examination and (C) combinations of a limited number of items. Whenever a red flag appeared in one clinical item, the examination (either full examination or selection of items, respectively) was marked suggestive of structural brain damage. We contrasted this information with the patient’s main diagnosis that was established after diagnostic work-up (independent variable, categorized into acute structural brain damage or other causes of CUE). Sensitivity and specificity of clinical tests were calculated using contingency tables. Sensitivity was defined as the ability of a clinical test (or a combination of clinical tests, respectively) to detect patients with acute primary structural brain pathologies (proportion of positive tests in affected patients). Specificity was defined as the ability of a clinical test (or a combination of clinical tests, respectively) to only detect such patients (proportion of negative tests in patients with other causes of coma). The degree of association between a positive neurological examination and underlying acute structural brain damage was assessed by calculating binary logistic regressions. Model quality and goodness of fit were evaluated by Nagelkerke’s (pseudo-) *R*^2^ and Hosmer–Lemeshow-Tests (HLT). *p* values below 0.05 were considered significant. We used IBM SPSS Statistics 26 (IBM, USA) for analysis.

## Results

We report on 853 emergency patients who were unconscious for unknown reasons at the time of examination in the ED and at least until all emergency diagnostics were completed (462 males, 391 females; median age 65, IQR 48–77 years.; median GCS 5, IQR 3–8).

### Full neurological examination

The full neurological examination including all items was 74% sensitive and 60% specific in detecting acute structural brain damage underlying CUE (*R*^2^ = 0.144, HLT not significant; Table 2). Given the suppressive effects of sedatives on neurological functions, we then separated 400 patients who had not been given sedatives at the time of examination (median GCS 7, IQR 5–9; 11 of them intubated; 142 of them with acute structural brain damage) from 453 patients who had received sedatives (median GCS 3, IQR 3–6; 188 of them with acute structural brain damage). Sedation was administered either for endotracheal intubation (299 patients), treatment of seizures or other reasons. In non-sedated patients (NSPs; *R*^2^ = 0.285, HLT not significant), sensitivity of the full neurological examination increased to 87% while specificity remained at 61%. In sedated patents (SPs; *R*^2^ = 0.067, HLT not significant), sensitivity was at 64% while specificity was at 58% (see Table 4).

**Table 2:** Contingency table showing sensitivity and specificity of abnormal findings in a full neurological examination with regard to detecting underlying acute structural brain damage in *n* = 853 unconscious emergency patients

	*n* = 853	Positive examinations	Negative examinations	
I	Primary cause, acute brain damage	244	86	74% Sensitivity
II	Primary cause, no acute brain damage	108	104	60% Specificity
III	Secondary cause	102	209	

Sensitivity = proportion of positive examinations among all patients with acute brain damage (I); specificity = proportion of negative examinations among all patients without acute brain damage (II + III)

### Single items of the neurological examination

In a second step, we investigated the diagnostic value of 10 single items that made up the full examination protocol in comparison between NSPs and SPs (Table 3). Gag reflex was not comparable between patient groups and was thus excluded from analysis as it had not been assessed in intubated patients. As expected, sensitivity of each single item was significantly lower in NSPs and SPs when compared to the full examination, ranging from 3 to 48% (NSPs) and 1% to 35% (SPs). However, specificity was high throughout, ranging from 80 to 99% in both NSPs and SPs. Five of the single items showed comparably high values for sensitivity: Asymmetrical pupil size (B) was 33% sensitive in NSPs and 35% in SPs. Abnormal gaze position (D) was 43% sensitive in NSPs but only 15% in SPs. Lateralized motor deficits (G) were 48% sensitive in NSPs, but only 7% in SPs. Likewise, asymmetrical muscle tone (H) was 34% sensitive in NSPs, but only 7% in SPs. Positive pyramidal tract signs (J) were 48% sensitive in NSPs and 21% in SPs. In a further analysis, we evaluated the single items by their statistical significance as covariates in a binary logistic regression model. Here, three items stood out: Asymmetrical pupil size (B) which was significant again both in NSPs and SPs, meningism (A) and abnormal gaze position (D) which were significant in NSPs only.

**Table 3:** Comparison of single items of a neurological examination in terms of sensitivity (Sens.) and specificity (Spec.) to detect underlying acute brain damage in unconscious patients as well as their statistical significance (*p*) within binary logistic regressions that used all ten items as covariates

		Non-Sedated Patients			Sedated Patients		
		*n* = 400			*n* = 453		
		Sens. [%]	Sens. [%]	*p*	Sens. [%]	Sens. [%]	*p*
A	Meningism	6.4	97.6	0.03*	2.7	99.0	0.14
B	Asymmetrical pupil size	**32.8**	89.3	0.01*	**35.1**	89.6	0.00*
C	Asymmetrical light reaction	10.0	95.4	0.82	5.0	96.0	0.34
D	Abnormal gaze position	**43.2**	82.9	0.00*	15.4	80.5	0.53
E	Asymmetrical vestibulo-ocular reflex	2.6	98.7	0.09	1.5	99.4	0.72
F	Asymmetrical corneal reflex	4.3	97.8	0.31	5.1	96.2	0.12
G	Asymmetrical motor function	**47.9**	88.8	0.13	7.5	89.8	0.62
H	Asymmetrical muscle tone	**33.7**	91.3	0.07	7.0	91.6	0.28
I	Asymmetrical tendon reflexes	13.3	95.5	0.69	3.7	94.9	0.97
J	Pyramidal tract signs	**48.4**	88.5	0.06	**21.3**	85.8	0.13

Bold script indicates sensitivity > 20%, stars indicate *p*-values < 0.05

### Combinations of select items

In a third step, we calculated the overall diagnostic value of two combinations of select items from the full examination protocol (Table 4). First, a combination of the five items showing the highest levels of sensitivity across NSPs and SPs: pupil size, position of gaze, motor function, muscle tone and pyramidal tract signs (B, D, G, H, J) plus pupillary reflexes to light (C; we added this despite its low sensitivity in the preceding analysis because when assessing pupil size using a flashlight, examiners regularly generate information on pupillary reflexes. It would have been absurd to actively disregard these findings). This 6-item variant was 82% (NSPs) or 62% (SPs) sensitive and 62% (NSPs) or 61% (SPs) specific to detect acute structural brain pathology underlying CUE.

**Table 4:** Comparison of three examination protocols (full, 6-item, 4-item) in terms of their ability to distinguish acute structural brain damage from other underlying causes in *n* = 853 unconscious patients (non-sedated, sedated, all)

	Non-sedated patients			Sedated patients			All patients		
	*n* = 400			*n* = 453			*n* = 853		
	Sens. [%]	Sens. [%]	CP [%]	Sens. [%]	Sens. [%]	CP [%]	Sens. [%]	Sens. [%]	CP [%]
Positive full examination	86.6	61.2	70.3	64.4	58.5	60.9	73.9	59.8	65.3
POSITIVE 6-item examination (B, C, D, G, H, J)	82.4	62.4	69.5	61.7	60.8	61.1	70.6	2.0	65.1
Positive 4-item examination (B, C, D, J)	77.5	66.5	70.5	58.5	63.1	61.2	66.7	64.8	65.5

*Sens.* sensitivity, *Spec.* specificity, *CP* percentage of correct predictions in binary logistic regressions

**Fig. 2:**
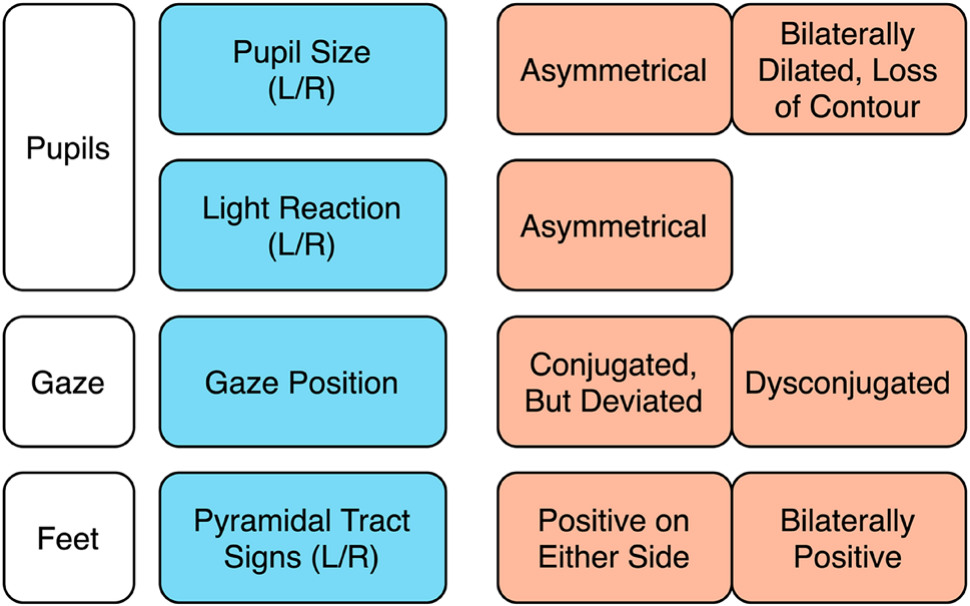
Quick 4-item examination protocol to be used as one component among many within an integrated diagnostic work-up of emergency patients presenting with CUE. Left: Items to be evaluated; right: possible results pointing towards acute structural brain damage

In a next step, we further reduced this 6-item combination by excluding motor function and muscle tone because the latter are the most laborious to examine reliably, especially in a critically ill emergency patient (B, C, D, J, Table 3, see also Fig. 2). This second, 4-item variant of the neurological examination was still 77% sensitive and 67% specific in NSPs and 59% sensitive and 63% specific in SPs (Table 4).

There was no difference in overall correct predictions between the 4-item and the 6-item variants, though values differed between NSPs (70%) and SPs (61%).

### Comparison of protocols

When comparing the three different examination protocols (Table 4) across all patients (independent of sedation), sensitivity declined slightly from 74% (full) to 71% (6-item) and 67% (4-item). Specificity, however, increased from 60% (full) to 62% (6-item) and 65% (4-item). However, percentage of correct predictions remained at 65% regardless of the examination protocol. The same pattern emerged in the sub-groups of NSPs and SPs, only at slightly higher (70% in NSP) or lower levels (61% in SP), respectively. In summary, the percentage of correct predictions was affected by sedation only but did not depend on the extent of the clinical examination.

## Discussion

This analysis of structured clinical examination data from patients presenting with CUE points out several important limitations of the neurological examination as a tool to detect the correct etiology in an emergency.

First, the full neurological examination had a low sensitivity and specificity to detect acute structural brain damage in unconscious patients. In our cohort, the percentage of overall correct predictions by clinical findings was only 65%, which is clearly insufficient given the severity of the clinical problem at hand. In statistical terms, predictions did not fit observations sufficiently which is reflected in an insignificant goodness of fit in binary logistic regression models. Second, the administration of sedatives, which happened in the emergency for various reasons and mostly outside the responsibility of the emergency physician in charge, further decreases the diagnostic value of the clinical examination. While this is hardly surprising, it is important to point out, that even in a sub-group analysis of non-sedated patients, the predictive value of the examination is below optimal standards. Third, the full neurological examination of unconscious patients was not associated with a higher sensitivity, specificity, or percentage of overall correct predictions compared to a concise examination protocol that only consists of four items and can be completed with little effort and almost regardless of specialist medical training in a minimal amount of time.

While several studies evaluated the prognostic value of results of parts of the clinical examination as integrated in the FOUR Score [20], data on the value of the neurological examination as a part of the diagnostic process in emergency patients with non-traumatic coma are scarce. However, Forsberg et al. reported focal neurological signs (defined as pupil asymmetry, Babinski reflex on one or both sides, asymmetry in motor response or deep tendon reflexes) in 24.6% of 633 patients with metabolic coma (also including patients with status epilepticus, seizures and postictal state) [21]. This is a lower percentage than in our cohort where 41% of patients without acute structural brain damage had focal signs (51% in category II and 33% in category III; see Table 2). This difference might be explained by the fact that not only neurologists had performed the examinations in their cohort but also Forsberg and colleagues analyzed medical records retrospectively.

There are several reasons that may explain the shortcomings of the neurological examination as a diagnostic tool in unconscious patients. Ideally, primary structural brain pathologies underlying disorders of consciousness should be clinically detectable by abnormal neurological findings. However, even in patients with unimpaired consciousness, the sensitivity of many clinical tests such as testing for meningism is well below 100%. A recent meta-analysis reported a sensitivity of 46.1% for nuchal rigidity in patients with suspected meningitis [22]. A Cochrane review indicated a sensitivity of 75.5% for the jolt accentuation of headache, which decreased to 65.3% when patients with impaired consciousness were included [23].

Further, significant abnormal findings in unconscious patients are also found in patients without acute brain damage and systemic disorders. Obviously, constricted pupils are caused by opioid intoxication more often than by isolated pontine damage. But maybe less obviously, the whole range of possible metabolic disorders (depending on their severity) may also cause asymmetrical neurological abnormalities that may be misleading [21, 24–29]. Moreover, neurological abnormalities may have been pre-existing and may thus falsely suggest acute structural brain damage. Not knowing a patient’s detailed past medical history, which is often the case early on in an emergency, may therefore also misdirect the course of management if major decisions are mainly based on clinical findings. Neurological abnormalities are thus generally not entirely specific for acute neurological causes.

Another problem is that many patients presenting with CUE have, in fact, more than just one underlying pathology that could explain the disorder. As reported earlier, one third of patients who were managed according to the same standard operating procedure were found to have multiple coma-explaining conditions [2]. For example, subdural hemorrhage may have led to asymmetrical motor dysfunction. However, if this clinical information alone determines further management, equally pressing and coexisting disorders such as acute hepatic failure following chronic substance abuse and now severe acute intoxication may go undetected.

Furthermore, there is a significant interobserver variability associated with neurological observations in general and specifically so in unconscious patients [30]. Subtle abnormalities are easily missed or underestimated while certain phenomena tend to be overestimated (e.g., minimal differences in pupil size, stiffness of the neck, spontaneous eye movements) or misread (e.g., directional vs. non-directional reaction to painful stimuli).

And not least, iatrogenic sedation (e.g., in recently intubated patients) may have masked neurological signs that should be present following a causal brain lesion. In our cohort, patients who eventually turned out to have acute structural brain damage were slightly more likely to be sedated at the time of examination (57% vs. 51% in patients without acute structural brain damage) and thus even more difficult to discriminate by clinical findings. However, in a time-critical emergency, clinicians need to focus and rely on robust decision-supporting tools that can be applied regardless of unswayable factors such as pre-hospital application of sedatives.

Calculating sensitivity and specificity for single items of a neurological examination is a somewhat artificial approach to determine their diagnostic value since these items usually only test for a specific dysfunction and not for any kind of brain damage. However, the approach is not too far from clinical reality, where examiners are faced with a variety of testing methods on the one hand and an even wider variety of conditions to be detected on the other hand. There is no single test that is sufficient and there are tests that are statistically insignificant on their own. However, there appears to be a selection of tests better suited to the problem than others as is reflected by their relatively higher sensitivity and, in fewer cases, statistical significance in regression models. This seems to be true for pupil size even regardless of sedation. Predefined answers recorded in the examination protocol applied in this study (Fig. 1) did not map the complete range of subtle, yet possibly meaningful findings in unconscious patients, resulting in a reduction of complexity. This was intended to adapt the protocol to the limited possibilities in a real-life emergency. Even for dedicated specialists, it is extremely difficult to perform and record an exhaustive clinical evaluation of an unconscious patient—whose status may or may not be influenced by iatrogenic sedation—in limited time and in a crowded emergency room.

In this large cohort of acutely unconscious patients presenting with the full range of possible underlying pathologies, the clinical neurological assessment proved not to be sufficiently reliable in finding the correct diagnosis. In an acute emergency, our data support a quick and simple neurological assessment instead of a lengthy examination (Fig. 2). It may be focused on the evaluation of pupils (size and reaction to light), gaze position and pyramidal tract signs (“pupils, gaze and feet”), that is to say the statistically more robust items. This short protocol is not intended to evaluate or quantify the level of unconsciousness. Rather, it is meant to support detecting the etiology of unconsciousness. It saves critical time while providing the same level of overall correct prediction.

In our analysis, the evaluation of motor function of the limbs (including assessment of muscle tone and tendon reflexes) does not relevantly improve sensitivity or specificity despite being considerably more work. Likewise, we left out assessment of meningism, vestibulo-ocular and corneal reflexes because they appeared with very low values for sensitivity. They add little to the diagnostic value of the examination protocol, most likely because they are either very likely to disappear in deeper stages of coma or particularly unreliable to assess. In an emergency that needs urgent ABC management, there is often little tolerance for the examiner, especially in the head and neck area of the patient. In addition, by being simpler and easier to reproduce, the proposed four-item protocol may help minimize interobserver variability that can result from varying qualifications of the personnel involved [31, 32].

To the best of our knowledge, this is the first study that prospectively assessed the value of a structured neurological examination in terms of identifying an acute structural brain damage in a large real-life cohort of patients with CUE. Nevertheless, the value of studies on routinely acquired clinical data and especially on clinical observations is associated with intrinsic limitations, the most substantial of which are interobserver variability and documentation bias. Further, we cannot exclude an examiner bias, since we did not analyze the effects of clinical experience of the neurologists performing the examination.

Although it is not a sufficient diagnostic tool, we are certainly *not* in favor of omitting the neurological examination. Next to its initial purpose of accurately assessing the level of consciousness, a full and thorough neurological examination is required in any unconscious patient as soon as circumstances allow. It is the most important monitoring tool for the individual patient’s recovery or decline and may trigger further diagnostics and guide treatment decisions in conjunction with all available information. In an acute emergency, however, less may be the same in terms of predictive value and maybe even more in terms of critical time being saved to save lives. From our data, we conclude that clinical neurological information on unconscious emergency patients should not solely determine clinical decisions but rather be seen as one of several complementary sources of information within a multimodal diagnostic approach to CUE. All CUE patients should receive a complete and thoroughly executed set of diagnostics as quickly as possible, embedded in a structured management routine including early brain imaging [19]. Further research thus needs to focus on optimizing the combination of clinical information and diagnostic investigations required to detect causes of CUE as quickly and reliably as possible.

## Data Availability

The data that support the findings of this study are available from the corresponding author upon reasonable request.
